# ‘I Battle With This Gambling Addiction Every Day’. Learning From Lived Experiences of Recovery to Guide Gambling Harm Prevention Strategies

**DOI:** 10.1111/hex.70719

**Published:** 2026-06-14

**Authors:** Simone McCarthy, Hannah Pitt, Paul Fung, Margaret Potter, Grace Arnot, Samantha Thomas

**Affiliations:** ^1^ Faculty of Health, Institute for Health Transformation Deakin University Geelong Australia; ^2^ Gambling Harm Lived Experience Experts Melbourne Australia

**Keywords:** gambling, health policy, health services, qualitative, recovery

## Abstract

**Background:**

Gambling harm is a significant public health issue affecting individuals, families and communities. In Australia, gambling is deeply embedded within everyday life, supported by the widespread availability of gambling products and industry marketing. This saturation has normalised gambling, obscuring its risks and creating barriers to recognising harm and seeking help. While clinical treatment pathways remain important, many individuals recover outside of formal services. There is increasing recognition that understanding and supporting recovery requires centring lived experience perspectives and adopting a broader public health and social determinants approach. This study sought to document gambling harm recovery journeys through lived experience.

**Methods:**

This qualitative participatory study used photo elicitation and in‐depth interviews with 13 adults in Victoria, Australia, who self‐identified as being in recovery from gambling harm. Participants were asked to capture photographs and reflect on key aspects of their recovery journey, including help and support seeking, facilitators and barriers to recovery and social and environmental influences. Reflexive thematic analysis was conducted collaboratively by researchers and lived experience experts to identify shared patterns of meaning.

**Results:**

Three key themes were developed: rebuilding identity and connection in recovery, navigating structural barriers and everyday triggers, and living with grief, loss, and ongoing vulnerability. Recovery pathways were nonlinear and shaped by individual, socio‐cultural, environmental and commercial determinants. Participants described stigma, the pervasive presence of gambling marketing, and ineffective regulatory protections as significant barriers to recovery. Facilitators of recovery included peer support, reconnecting with meaningful activities and developing strategies to avoid gambling environments.

**Conclusion:**

Findings reinforce the need to move beyond individual‐focused harm prevestrategies towards systemic reforms that address the commercial and environmental determinants of gambling harm that impact recovery efforts. Strengthened regulation, expanded access to peer‐led and community‐based supports, and greater integration of lived experience into policy and service design are critical for supporting recovery. Participatory methods offer valuable opportunities to capture the complexity of recovery journeys and inform public health responses.

**Lived Experience Contribution:**

People with lived experience of gambling harm were employed as researchers on this study. They contributed to the study design, data collection and interpretation, co‐authoring of the academic article and dissemination of the findings.

## 1 Introduction

Gambling harm is a major public health issue, with significant consequences for individuals, families and communities across Australia and internationally. It has been broadly defined as ‘any kind of harm or distress arising from, or caused or exacerbated by, a person's gambling’ [1]. More contemporary definitions expand on this by highlighting the multiple dimensions of harm. Marionneau et al. [2] describe gambling harm as encompassing health, psychological, relationship, financial, cultural, work and crime‐related issues, with harms often persisting beyond the gambling activity or extending across generations. Similarly, Hilbrecht et al. [3] define harmful gambling as any repetitive gambling that results in recurring negative consequences, not only affecting the individual but also their families, social networks and communities. These definitions highlight that gambling harms extend beyond financial losses, including mental health challenges, relationship breakdowns, reduced workforce participation and broader societal costs [4].

Australia, often referred to as the ‘gambling capital of the world’ [5], has been described as having a deeply entrenched culture of gambling [6]. Australians lose approximately $32 billion annually on a range of different forms of gambling, with electronic gambling machines (EGMs) accounting for the majority of losses, and online wagering continuing to grow [7]. The social cost of gambling harm has been estimated at several thousand dollars per affected individual [8], with Victoria alone facing a burden of around $14.1 billion per year [4]. Notably, research shows that the majority of harm is attributable not only to people experiencing severe levels of harm but also to those experiencing low to moderate levels of harm [9, 10, 11].

Historically, gambling policy and treatment in Australia have focused on individual responsibility responses, framed largely through a harm minimisation model that promotes ‘responsible gambling’ [12, 13, 14]. However, this paradigm has been criticised for neglecting the broader structural drivers of gambling harm—including the strategies and tactics used by the gambling industry to promote products and prevent regulatory reform [15, 16, 17, 18]. In response, there is increasing recognition that gambling harm must be approached as a public health issue, arising from the interaction between a range of individual, socio‐cultural, environmental, commercial and political determinants [3, 19]. Similar patterns have been observed across other health‐harming industries such as alcohol and tobacco, where corporate practices, including marketing, product availability and political influence, shape environments that increase health risks [20, 21, 22]. A public health approach emphasises the need for upstream interventions, such as regulation of gambling products, significant restrictions on marketing and environments designed to prioritise community wellbeing over gambling product accessibility [23, 24]. Despite widespread support for public health approaches, government approaches to gambling harm remain largely focused on downstream individualised approaches (such as informed choice and help‐seeking campaigns) rather than structural reforms of the gambling industry, its products and promotions [19].

The normalisation of gambling further complicates efforts to prevent and reduce harm. Gambling in Australia is widely perceived as a normal part of cultural and social life [6, 25]. This normalisation is driven by widespread accessibility of gambling products in community and digital spaces, aggressive commercial marketing strategies (including personalised inducements and promotions), the integration of gambling with sport and leisure activities, and positive portrayals of gambling in traditional and social media spaces [26, 27, 28, 29]. Research has demonstrated that these promotional activities contribute to the social and cultural acceptance of gambling—particularly in young people—and reinforce the perception that gambling is a harmless, fun entertainment activity [30, 31]. The pervasiveness of gambling in Australian (and other) cultures creates significant challenges for those who have experienced gambling harm. Research suggests that the normalisation of gambling acts as a barrier to recognising harm, seeking help and maintaining recovery [32, 33].

Recovery from gambling harm is not simply an individual journey but is shaped by social, environmental and commercial contexts. Traditional models often equated recovery with abstinence or the clinical absence of gambling disorder [34]. However, contemporary frameworks emphasise broader conceptualisations that incorporate improvements in wellbeing, personal empowerment and meaning‐making [35, 36, 37]. Recovery may involve reduced gambling engagement, rebuilding relationships, addressing co‐occurring health issues and improving mental health [35, 38]. While clinical treatment remains important [39], research consistently shows that only a small proportion of people experiencing gambling harm engage with formal services [40, 41]. Barriers include stigma, shame, denial, limited service access and financial hardship [42, 43]. Given these barriers, natural or self‐directed recovery pathways are important to recognise. Many individuals recover through informal support networks, lifestyle changes and self‐help strategies without clinical intervention [44]. Arts‐based approaches, such as photography, art and music therapy, have emerged as valuable non‐clinical recovery supports, particularly for underrepresented groups [45, 46]. These approaches offer alternative avenues for meaning‐making, emotional processing and identity reconstruction. While there is a growing body of research involving people with lived experience of gambling harm [47, 48, 49, 50], studies exploring recovery journeys have predominantly relied on interviews or surveys. Visual methods such as photo elicitation have been used far less frequently in gambling harm research.

Lived experience perspectives are critical for understanding gambling harm prevention and recovery in a nuanced way. Across health sectors, integrating lived experience into research, policy, and practice has improved relevance, effectiveness and equity [51, 52]. In gambling research, Lived Experience Experts (LEX) have highlighted systems gaps, advocated for regulatory reforms, and brought to light the ongoing impacts of stigma and inequity [48, 53]. Research has demonstrated the value of lived experience expertise in strengthening responses to gambling harm, while warning that involvement risks becoming tokenistic if people are included without meaningful influence over decision‐making [48, 49]. Understanding the complex and diverse recovery journeys from gambling harm requires centring the voices of those who have lived it. The research therefore aimed to document recovery journeys from the perspectives of those with lived experience of gambling harm. Drawing on a public health approach, this study aimed to amplify lived experience perspectives to explore the barriers, facilitators and commercial influences that shape recovery and the lessons that can be learned for public health prevention initiatives and support services. The study was guided by three research questions:

1. What are the individual, socio‐cultural, environmental and commercial determinants that may influence the recovery journeys of those impacted by gambling harm?
2. How do these determinants create facilitators or barriers to recovery?
3. How can community participatory methods such as photo elicitation be used to provide a more holistic understanding of recovery journeys?

## 2 Methods

### 2.1 Approach

This study was guided by a public health approach to gambling recovery [23], underpinned by a social determinants of health framework [54] and a commitment to embedding lived experience expertise [48]. A public health lens enabled a broader analysis of recovery, recognising that individual health outcomes are shaped by social, environmental and commercial factors [23, 55]. Applying this perspective directed attention to structural barriers and enablers of recovery, such as access to support services, socioeconomic inequality and the normalisation of gambling. While the study was guided by a public health approach, we recognise that lived experience perspectives are diverse and that perspectives on recovery and harm may vary across contexts and do not represent a single or uniform view. Lived experience engagement was embedded across the research process, ensuring that those directly affected by gambling harm shaped the study's design, analysis and communication of findings. The research team comprised four public health academics with experience in gambling research and two researchers with lived experience of gambling harm. This combination of academic and lived experience expertise enabled both insider and outsider perspectives within the research process [56]. Throughout the study, the team reflected on how our backgrounds, expertise and experiences may have shaped the interpretation of the data, while remaining attentive to the diversity of perspectives represented within participants' accounts.

Greater than low‐risk ethical approval was received from Deakin University prior to data collection commencing.

### 2.2 Embedding Lived Experience

LEX researchers were employed as researchers on the project and contributed across all stages of the project, including study design, participant recruitment, data analysis, paper writing and dissemination activities. Their involvement aligned with established frameworks for lived experience engagement in gambling research [48, 53], which emphasise meaningful participation, capacity building, self‐determination and independence. LEX researchers received training in research ethics, qualitative interviewing and data interpretation to support their involvement. Their expertise enhanced the project's reflexivity and ensured that findings were grounded in lived realities rather than the researcher's assumptions.

### 2.3 Sample and Recruitment

A qualitative, participatory research design was employed using photo elicitation and semi‐structured interviews. Thirteen Victorian adults were recruited between April and October 2024 via purposive and snowball sampling methods. Eligibility criteria included being aged 18 years or older, residing in Victoria, self‐identifying as in recovery from gambling harm, being comfortable discussing their recovery journey, and having access to a device to capture photographs. Recruitment strategies included outreach through professional networks, social media and distribution of flyers through gambling support groups.

### 2.4 Data Collection

Photo elicitation was selected as a participatory method that enables participants to represent and interpret their experiences through images and discussion, supporting the co‐creation of knowledge between participants and researchers [57, 58]. This approach draws on participatory traditions in qualitative research that seek to redistribute power within the research process and centre the perspectives of people with lived experience. Photo elicitation has been widely applied in health promotion research and addiction recovery studies [59, 60], offering opportunities to challenge traditional researcher‐participant power dynamics and highlight marginalised perspectives. Participants were asked to capture at least five photographs reflecting key aspects of their recovery journey, focusing on themes aligned with the study's research questions, including help and support seeking, facilitators and barriers to recovery, and social and environmental influences. Participants then discussed these in individual interviews, where the photos served as prompts to enrich narrative accounts. The request for five photographs served as a guide rather than a strict requirement, and participants shared varying numbers of images depending on what they felt best represented their experiences. Participants were given approximately 2 –4 weeks to collect their photographs, with flexibility to allow more or less time depending on participants' needs. During this period, the researcher checked in with participants to see how they were progressing and to arrange a time for the interview. Interviews were conducted by an experienced qualitative researcher either via Zoom or face‐to‐face, depending on participant preference. Interviews lasted approximately 90 min and used the photographs as prompts to elicit deeper reflections and narratives. The interview schedule was semi‐structured, allowing participants to shape the direction of the discussion. While the aim was to use these images to encourage action on policy and practice, the analytic process was conducted by the research team, rather than collectively by participants, which differentiates this approach from photovoice [61]. Participants received a $200 voucher in recognition of the time and effort involved in taking photographs and contributing to the interview, acknowledging their valuable role in the research.

### 2.5 Data Analysis

Interviews were transcribed by a professional transcription company, and participant photographs were embedded within the transcripts to retain contextual meaning. Braun and Clarke's [62] Reflexive Thematic Analysis guided the analysis of qualitative data. This first involved familiarising oneself with the data from the one‐on‐one interviews, examining the photographs, correcting transcripts for accuracy and embedding the participant images into transcripts where they were discussed. This helped preserve the context and meaning attached to each image during analysis. Interview transcripts were then uploaded into the qualitative data management software package NVivo. Transcripts were analysed individually, with initial coding guided by the research questions and structure of the interview schedule. This provided a practical entry point for coding while remaining open to new insights. As analysis progressed, codes were refined and re‐organised to generate patterns of meaning that moved beyond the original interview structure. Initial themes were then reviewed across the dataset and grouped into broader thematic categories. The research team re‐examined the photographs during this process to ensure they were appropriately positioned within the developing thematic structure. Final themes were collaboratively reviewed, named and defined through regular team discussions, with LEX researchers actively involved to ensure interpretation was reflexive and grounded in lived experience. All the photographs were also organised according to the final thematic structure and were used to visually illustrate key aspects of the findings in the final write‐up. Identifying details were removed from photographs included in the manuscript to maintain participant anonymity. The results were written using participant quotes to illustrate the findings. Each participant was assigned a numerical label (Participant 1–13) to maintain anonymity while allowing readers to follow individual narratives throughout the manuscript. An additional table of illustrative quotes mapped to the themes and subthemes presented in this paper is provided in Supporting Information S1.

## 3 Results

### 3.1 Participant Demographics

The demographics of the 13 individuals who participated in the study are presented in Table 1. The majority of participants were aged 55+ years (*n* = 8), with four participants aged 35–54 (*n* = 4), and one participant aged 18–34 years. A higher proportion of males (*n* = 8) participated in this study compared to females (*n* = 5). Most participants had an education level of Year 12 or below (*n* = 8). Most participants were either employed full time (*n* = 5), or retired (*n* = 5). Across the sample, participants provided a total of 122 photographs, ranging from 2 to 31 photographs per participant (mean = 9.38).

**Table 1:** Participant characteristics (*n* = 13).

Demographic	Number
Age	
18–34	1
35–54	4
55+	8
Gender	
Male	8
Female	5
Highest level of education	
Did not complete high school	5
Completed high school	3
Certificate I, II, II, IV	1
Diploma/advanced diploma	3
Bachelors degree	1
Employment status	
Retired	5
Working full‐time	5
Working part‐time/casually	1
Unemployed	1
Volunteer	1

Three themes were constructed from participant interviews.

### 3.2 Theme One: Rebuilding Identity and Connection in Recovery

**Figure 1:**
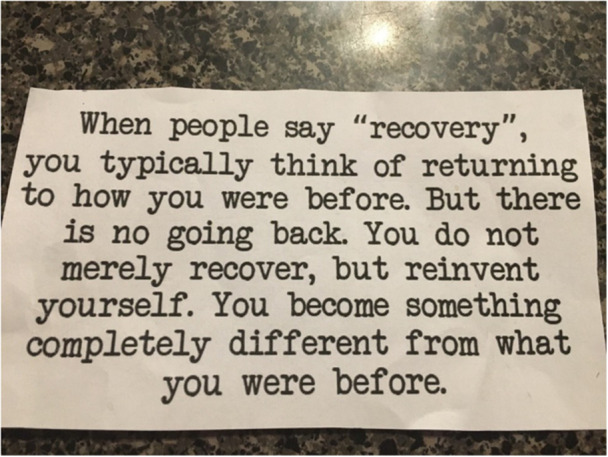
Identity in recovery.

Participants in this study described recovery as a deeply personal process of rebuilding their lives and reconnecting with the people, activities and places that mattered most to them. Recovery involved rediscovering value in themselves, developing new life goals and surrounding themselves with supportive relationships that had been lost or neglected during periods of gambling harm. Several participants reflected on finding a renewed sense of self after years of feeling broken, isolated, or controlled by gambling. Reconnecting with former interests, spending time in nature and developing new roles and routines were common pathways through which participants began to rebuild their lives. As one participant explained, the photograph shown in Figure 1 represented how she understood recovery as a process of identity change:

You do not merely recover, but you reinvent yourself. I live by that, and I even say, what do I want to be now?

—Participant 7

**Figure 2:**
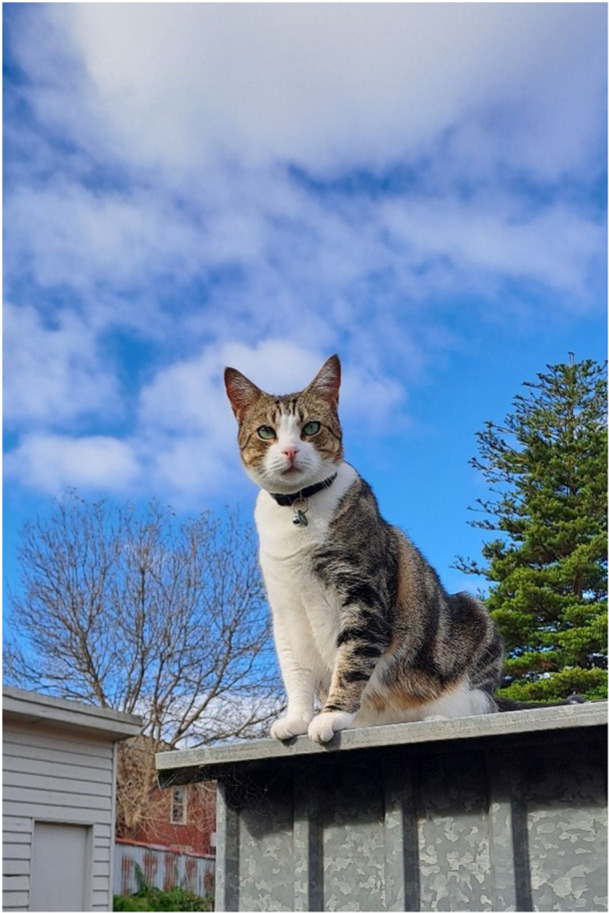
Connection in recovery.

Participants spoke about the importance of belonging, particularly to groups that provided understanding and unconditional support. Family connections played a significant role in recovery narratives. Gifts, shared moments, and acts of care were powerful symbols of renewal. As one participant recounted, ‘it's just about new beginnings, being brought back to life and the colour, the colour, you know, being brought back inside my soul’. Through these relationships, participants described rebuilding trust and reconnecting with sources of stability and support. For some, this came through relationships or peer support programmes. Some participants described supporting others who were experiencing gambling harm, drawing on their own experiences to provide encouragement, share insights and help others navigate recovery. For others, the sense of belonging was found through bonds with family members or animals that offered consistent emotional safety. Figure 2 illustrates how connection with her cats provided comfort and stability during recovery:

I take [the cats] out each day just for an hour or so, and just sit there and chill with them. I don't have a desire to be anywhere else. You know, ‘Oh, it would be better to be at the pokies than this.’ No, they mean everything to me.

—Participant 9

Participants also described the importance of engaging in meaningful activities as part of rebuilding their lives after gambling harm. These included reconnecting with hobbies, spending time in nature, rebuilding relationships with family and friends, and, in some cases, supporting others experiencing gambling harm. Such activities helped create structure in daily life and reinforced a sense of identity beyond gambling. Natural spaces, especially the ocean and nature, were described as restorative. One participant said that the ocean had become her happy place:

The sea and the ocean is my happy place. Whenever I'm down, whenever I've got any problems in life, I go and sit by the ocean, and that just calms me down.

—Participant 4

**Figure 3:**
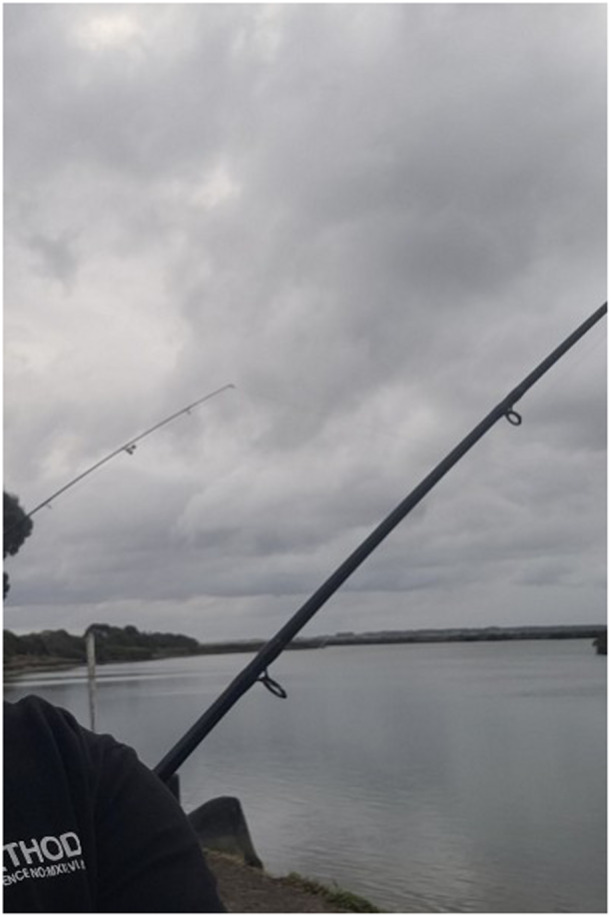
Reconnecting with hobbies, places and routines.

Similarly, participants spoke about reconnecting with hobbies that had once been important to them. One example, presented in Figure 3, was returning to fishing, a pastime that this participant had enjoyed in childhood:

I looked back at what I really enjoyed when I was younger. One of those things was fishing. So I started actively participating in fishing.

—Participant 4

### 3.3 Theme Two: Navigating Structural Barriers and Everyday Triggers

**Figure 4:**
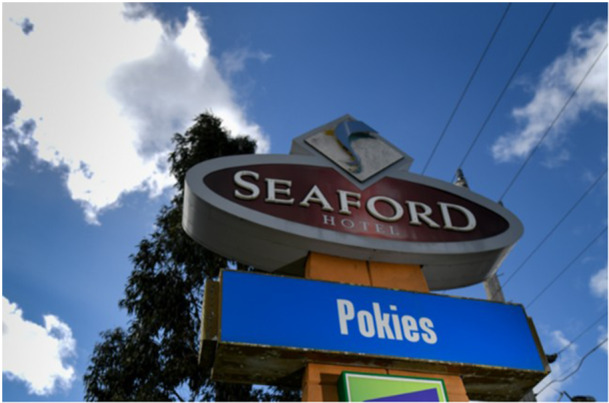
Saturation of gambling advertising.

Participants consistently highlighted the challenges of navigating recovery within an environment that remained saturated with gambling advertisements, promotions and venues/products. Despite their efforts to rebuild their lives, participants described being continually exposed to gambling triggers in everyday settings. These structural conditions meant that recovery often involved actively managing or avoiding situations where gambling was normalised or highly visible. Participants described avoiding particular areas where they knew there were gambling venues, limiting exposure to sporting matches, or restructuring their daily routines. The omnipresence of gambling venues and signage in local communities was particularly challenging. One participant used the photograph in Figure 4 to explain the overwhelming nature of these everyday triggers:

Driving past anywhere and everywhere you go in Victoria the pokies signs are there. Each sign you drive past is a trigger.

—Participant 2

**Figure 5:**
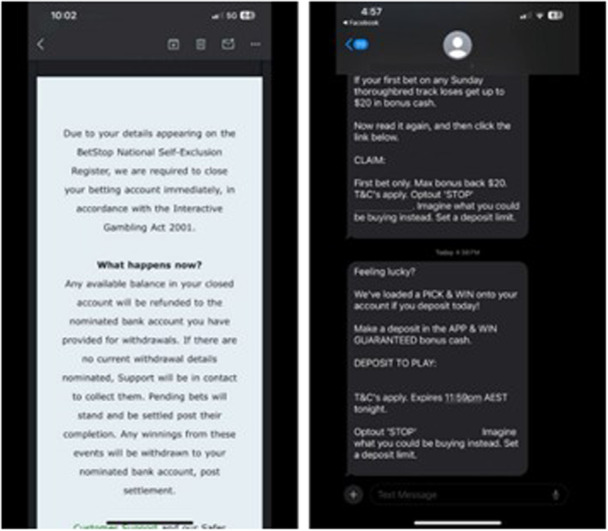
Ineffective mechanisms to stop promotions.

The frustration of constant exposure to gambling was compounded by what participants perceived as ineffective regulatory and policy protections. Several participants shared experiences of self‐excluding from gambling platforms only to continue receiving promotional messages. Despite signing up for BetStop, the national self‐exclusion programme for online forms of gambling such as sports betting, some participants reported ongoing direct marketing from betting agencies, which undermined their attempts to distance themselves from gambling environments. Figure 5 depicts how Participant 12 experienced ongoing advertising despite self‐exclusion:

Even though I had BetStop, the ads still popped up. It's a disgrace… It's a little reminder kind of thing. Like a slap in the face.

—Participant 12

**Figure 6:**
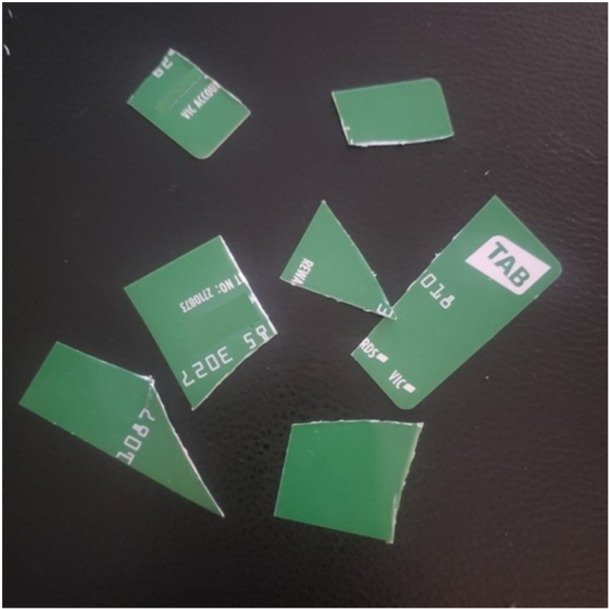
Strategies to avoid gambling.

Participants described practical strategies to navigate these environments and protect their recovery. These included altering their routes and limiting exposure to gambling environments, and removing reminders of past gambling behaviours. Some also described symbolic acts of defiance, demonstrated in Figure 6, where cutting up gambling venue membership cards or formally self‐excluding from venues and online platforms were seen as important steps in reclaiming control:

Cutting up the card is saying, ‘No, I'm not going to gamble anymore’.

—Participant 10

**Figure 7:**
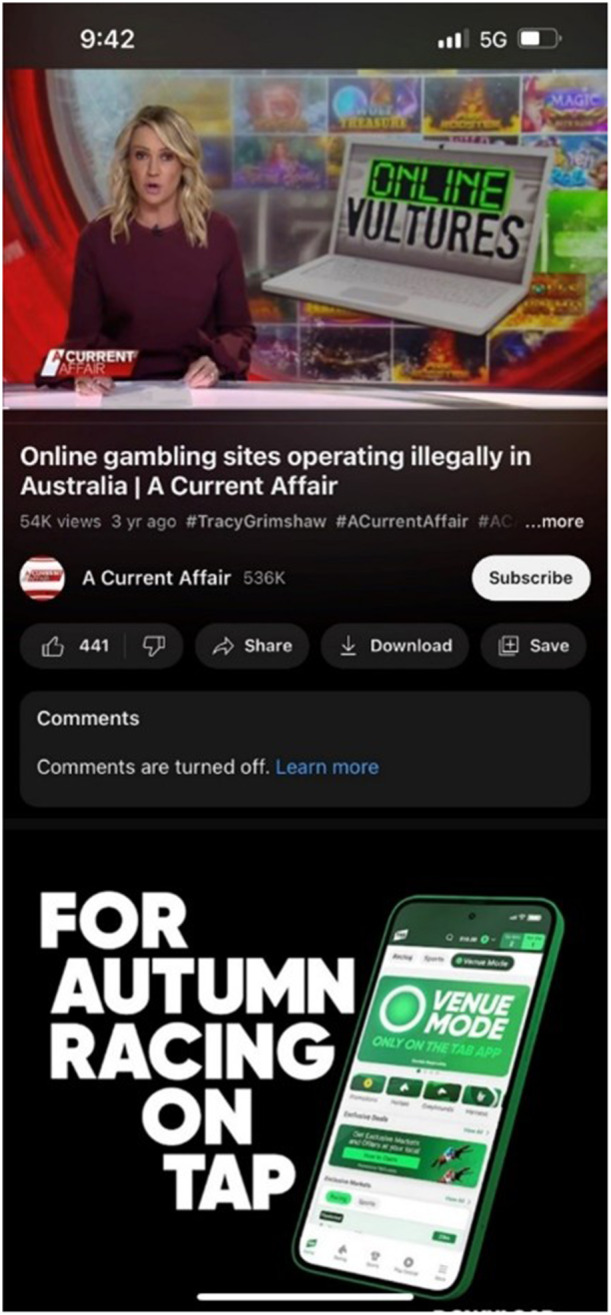
Seeking recovery resources.

Participants highlighted the importance of support services, prevention initiatives and policy reform in both reducing gambling harm and supporting recovery. Many described actively seeking out support at different stages of their recovery journey, emphasising the importance of accessible and adequately resourced services that could respond to diverse needs. For some, formal counselling was described as particularly valuable in helping them process the impacts of gambling harm and rebuild their lives. At the same time, participants noted that gaps in service availability and consistency, especially when coupled with financial barriers, made accessible support more difficult. While support services were seen as important, participants also recognised that individual recovery efforts could only go so far in the face of broader systemic drivers of harm. Many expressed disillusionment at the pervasive nature of the problem. Many expressed frustrations at the pervasive nature of gambling promotion and availability. One participant captured Figure 7 when seeking information about gambling, and reflected on how visible gambling advertising remained even when attempting to access recovery resources:

You go onto YouTube to look up gambling reform, and gambling ads will come up at the end of it. It's putrid.

—Participant 3

### 3.4 Theme Three: Living With Grief, Loss and Ongoing Vulnerability in Recovery

Recovery was not experienced as a linear or complete process but as an ongoing process of living with loss, grief and vulnerability. Participants described profound emotional impacts that lingered long after they had stopped gambling. One participant reflected on the impacts of falling into depression and suicidal ideation as a result of his gambling.

I've got a lot of scars here on both arms from trying to commit suicide… that's from doing all my money, depression through gambling.

—Participant 1

**Figure 8:**
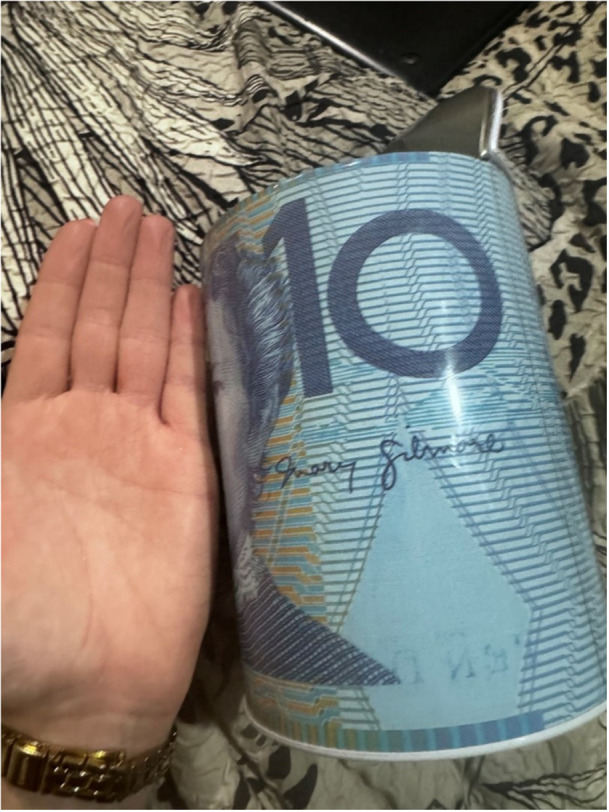
The emotional toll of gambling.

Material losses were also vividly described, symbolising the tangible costs of gambling harm. Participants spoke about losing homes, relationships and items that represented personal achievement. One particularly discussed the forced sale of a car that embodied independence and hard work: ‘I had to sell this car… it was the only thing I had that I'd bought for myself’. Alongside these tangible and emotional losses, participants described continuing to grapple with shame, regret and the challenge of rebuilding trust in themselves and with others. Some participants captured this experience symbolically, such as through Figure 8 of an image of a money box representing secrecy and desperation:

That symbolises desperation… the secrecy, because you don't want to tell anybody you did that, do you?

—Participant 3

**Figure 9:**
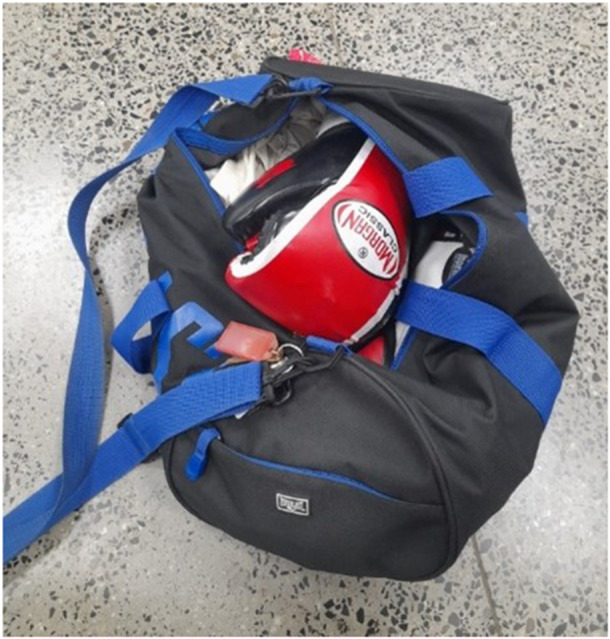
Lasting effects of gambling harm.

Others reflected on how the lasting effects of gambling harm continued to shape their everyday lives. One participant described how they still battle gambling thoughts daily, despite outward appearances of recovery. They used an image of a gym bag (Figure 9) to symbolise these thoughts:

Now I still pull the doona over my head because I battle with this gambling addiction everyday. At least I go ‘Oh well, you trained hard yesterday, you can sleep in’, right?

—Participant 5

**Figure 10:**
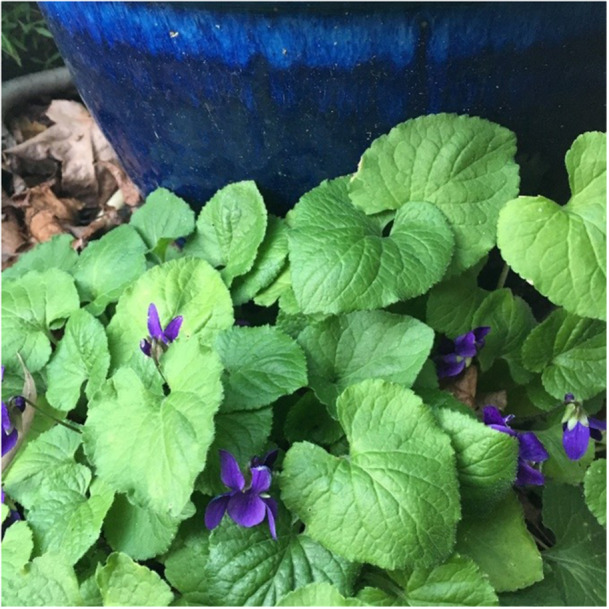
Turning to nature to heal.

At the same time, participants described developing ways to live alongside these experiences and continue moving forward. Many spoke about turning to nature, art and small daily practices to help create moments of calm and perspective. One participant captured Figure 10 and spoke about the symbolic power of flowers in representing resilience and beauty amidst grief:

They represent the tininess, the tiny beauty that's everywhere.

—Participant 7

For all participants, recovery was about more than abstinence. Participants described the recovery process as learning to live with what had been lost, finding new meaning in the present, and acknowledging that vulnerability would remain a part of their lives. Within that acknowledgement was also hope, reflected in participants' efforts to rebuild their lives while recognising the lasting impacts of gambling harm:

Recovery is never too late. This is me now. It's never too late to be what you might have been. That gives me hope. I'm not going to waste my time looking back, thinking about what I've wasted. I will do it now, I'll do what I can now, in whatever limited way I can do it.

—Participant 7

## 4 Discussion

The research aimed to document recovery journeys from the perspectives of those with lived experience of gambling harm. The findings raised three points for discussion in relation to the research questions.

First, participants' recovery pathways were influenced by a wide range of factors extending beyond individual behaviours. Recovery was not a linear or solely treatment‐driven process but was profoundly shaped by broader structural and social conditions. Consistent with public health perspectives on gambling [23], participants' experiences highlighted that mental health challenges, financial stress, stigma and normalisation of gambling within families and communities played critical roles in shaping engagement with gambling and barriers to stopping. Although the analysis was guided by a public health perspective, participants' accounts reflected diverse experiences and perspectives on gambling harm and recovery. Commercial influences were especially important, with participants reporting that the pervasive presence of gambling marketing and ease of access to gambling products created ongoing challenges to recovery. These findings align with evidence that recovery is often disrupted by socio‐environmental conditions that reinforce gambling behaviours [3, 37]. While some participants accessed formal services such as counselling or self‐exclusion programmes, many also drew on informal strategies, including peer support and reconnecting with meaningful activities. These diverse recovery strategies reflect broader research in addiction recovery, which recognises the importance of non‐treatment pathways [63, 64]. Importantly, these findings contribute to emerging literature on the commercial determinants of gambling recovery [65, 66], emphasising that recovery initiatives must account for the environments and commercial practices that individuals navigate daily. Flexible, culturally safe and contextually aware services are needed to support the complexity of recovery experiences. These results point to the need for health services and treatment providers to move beyond narrow clinical models. Gambling support interventions should be designed to accommodate diverse pathways, be culturally safe and flexible, and address the wider social and commercial conditions that exacerbate gambling harm. Embedding these principles within service delivery would strengthen recovery pathways and reduce inequities in access to support.

Second, participants described a range of facilitators that supported their recovery journeys. Peer support was especially valued, providing a safe environment where individuals could share experiences, foster hope and learn practical coping strategies. This is consistent with evidence from other areas of addiction research showing that peer‐led initiatives play a vital role in enhancing recovery and building resilience [67]. Recent gambling research has also highlighted how people with lived experience may engage in peer support, advocacy and community activities that provide structure and meaning within recovery [49]. Counselling services and strong family relationships also facilitated sustained engagement in recovery, although access to these supports was not uniformly available. At the same time, substantial barriers to recovery were reported. Participants criticised the ineffectiveness of self‐exclusion systems, describing how these measures were easily bypassed and inadequately enforced. This reflects broader concerns about the limitations of self‐exclusion programmes [68, 69, 70]. Participants also highlighted the persistent saturation of gambling marketing, even after self‐exclusion, noting that aggressive promotional strategies by the gambling industry continued to undermine their recovery efforts. This aligns with research characterising industry marketing tactics as predatory, particularly toward individuals at risk [71]. These findings highlight the need for stronger policy action. Placing the burden on individuals to manage their recovery in environments saturated with gambling opportunities and promotions places undue responsibility on those experiencing harm and risks reinforcing the stigma around seeking help. A more effective approach requires governments to take stronger action on the commercial determinants of gambling harm through regulatory and policy reforms. This includes reducing the availability of products, eliminating gambling marketing in community and digital spaces, and shifting accountability from individuals to the gambling industry and those who financially benefit from gambling. Governments also have a critical role in ensuring that accessible and adequately resourced support systems are available for people experiencing harm, recognising that recovery is shaped by the broader environments in which gambling occurs. Adopting a comprehensive public health approach that addresses the commercial and environmental drivers of gambling [23] is critical for creating services that genuinely support recovery and prevent future harm.

Lastly, the use of photo elicitation as a participatory method proved critical in providing a more holistic and nuanced understanding of gambling harm recovery. While a growing body of research has explored gambling harm from lived experience perspectives [47, 48, 49, 50], studies examining recovery journeys have largely relied on interview‐based methods. Visual methods such as photo elicitation have been used in addiction and recovery research to explore lived experiences of recovery, including studies of alcohol [59] and gambling [60]. These approaches have also been recognised for their potential to rebalance traditional researcher–participant relationships by enabling participants to represent their experiences visually and contribute more actively to the research process [72]. In this study, visual storytelling allowed participants to communicate complex emotional experiences, to highlight the interplay between individual, environmental and commercial factors, and to articulate both vulnerabilities and strengths. This aligns with findings from arts‐based participatory research, which emphasise that visual methods can deepen engagement, empower participants and disrupt traditional researcher–participant power dynamics [61, 72, 73]. Embedding participant photographs into the analysis also enabled the co‐creation of knowledge, ensuring that interpretations remained grounded in participants' lived realities [57, 58]. By applying photo elicitation to explore recovery journeys from gambling harm, this study adds a visual participatory perspective to the growing body of lived experience research in this field. Importantly, participants' photographs were not only reflections on personal recovery but were also intended to communicate lived experience perspectives to broader audiences. Future gambling harm research should continue to integrate participatory, arts‐based approaches, especially when seeking to amplify the voices of those most affected. Ensuring that lived experience is not only included but meaningfully centred in research, policy and practice is critical for developing responses that address the full complexity of gambling harm and recovery. For public health, this highlights the importance of participatory and creative methodologies in producing knowledge that is both rigorous and relevant. By centring lived experience and challenging traditional power hierarchies, such approaches can strengthen the design of interventions, improve policy responsiveness and support lived experience voices in influencing public debate and policy responses to gambling harm.

## 5 Limitations

This study was conducted in Victoria, Australia, and findings should be interpreted in light of the specific regulatory, cultural and environmental gambling contexts of this state. While the study sample was small, consistent with qualitative research approaches, the use of in‐depth interviews alongside photo elicitation methods contributed rich, detailed insights into the recovery from gambling harm. The sample skewed towards older participants, suggesting that younger people may face different challenges or be less likely to disclose their experiences, and future research should consider ways to engage these groups. Participant ethnicity was not collected as part of this study, limiting the ability to consider how experiences of gambling harm and recovery may differ across diverse cultural communities. This study also covered a range of gambling products and environments; future research could explore differences across specific settings to inform targeted interventions and policies.

## 6 Conclusion

This study highlights that recovery from gambling harm is a deeply personal yet socially and structurally influenced journey. Participants' experiences emphasised the importance of rebuilding identity and connection, navigating environments saturated with gambling triggers, and living with the ongoing emotional impacts of harm. Recovery was shaped not only by individual motivation but also by broader socio‐cultural, environmental, and commercial determinants that created both facilitators and barriers. These findings reinforce the need for flexible, culturally safe, and structurally responsive support systems that move beyond individual‐focused models of harm reduction. The study also demonstrates the value of participatory methods such as photo elicitation in capturing the complexity of recovery journeys. By centring lived experience, this approach offered a more holistic understanding of the challenges and opportunities encountered in recovery and highlighted the importance of lived experience voices in informing public debate and policy responses to gambling harm. Strengthening community‐based support, embedding lived experience into policy development, and prioritising structural reforms that address the commercial determinants of gambling harm are critical steps toward creating sustainable recovery pathways.

## Author Contributions

**Simone McCarthy:** conceptualisation, formal analysis, funding acquisition; writing – original draft, writing – review and editing, data curation. **Hannah Pitt:** conceptualisation, data curation, formal analysis, funding acquisition, writing – original draft, writing – review and editing. **Paul Fung:** funding acquisition, data curation, formal analysis, writing – original draft, writing – review and editing. **Margaret Potter:** formal analysis, data curation, funding acquisition, writing – original draft, writing – review and editing. **Grace Arnot:** data curation, formal analysis, writing – original draft, writing – review and editing. **Samantha Thomas:** conceptualisation, funding acquisition, writing – original draft, writing – review and editing, formal analysis, data curation and senior author of the manuscript.

## Ethics Statement

Ethics approval was provided by the Deakin University Human Research Ethics Committee (2023‐333).

## Conflicts of Interest

S.M. has received funding for gambling research from the Australian Research Council, Victorian Responsible Gambling Foundation, VicHealth, NSW Office of Responsible Gambling, Department of Social Services, ACT Gambling and Racing Commission, and Deakin University. M.P. has been employed on a range of projects relating to gambling by Gamblers Help. P.F. has been employed on projects relating to gambling by the Victorian Responsible Gambling Foundation; Banyule Community Health; Respin speakers bureau; Self Help Addiction Resource Centre; Three Sides of the Coin; Brimbank City Council; The Alliance for Gambling Reform; and EACH. H.P. has received funding for gambling research from the Australian Research Council, Victorian Responsible Gambling Foundation, VicHealth, NSW Office of Responsible Gambling, Department of Social Services, ACT Gambling and Racing Commission, and Deakin University. She sits on an advisory committee for the Alcohol, Tobacco and Other Drugs Association ACT. G.A. has received funding for gambling research from the ACT Gambling and Racing Commission and VicHealth. ST has received funding for gambling research from the Australian Research Council, ACT Gambling and Racing, Department of Social Services, VicHealth, Victorian Responsible Gambling Foundation, Healthway, NSW Office of Responsible Gambling, and Deakin University.

## Supporting information

Supporting File.

## Data Availability

The interview data and photographs are not publicly available as the individuals participating in this survey did not consent to their data being shared beyond the research team.
