# Supplementary material for: ‘I Battle With This Gambling Addiction Every Day’. Learning From Lived Experiences of Recovery to Guide Gambling Harm Prevention Strategies
Source: Health Expect. 2026 Jun 14;29(3):e70719. doi: 10.1111/hex.70719 (PMC13264681; doi:10.1111/hex.70719)
Supplement: Supplementary file 1 — Supporting File. [file HEX-29-e70719-s001.docx]

**Supplementary file: Table of quotes**

| **Subtheme** | **Quote** |
| --- | --- |
| **Theme one: Rebuilding identity and connection in recovery** | |
| Identity and self worth | “I had to find a new way of living, a new way of thinking about it, and I came up with a mantra, strength and courage, it will take all my strength and all my courage to defeat this overwhelming addiction, this overwhelming need to gamble on the pokies, to gamble on anything, by then I was gambling on everything, gambling on the pokies. I need strength and I need courage to do it, to stand up to it, say no more.” – Participant 13  “I have a lot of self worth now, and I've gone through a lot and I've done a lot of things I'm not proud of too, a lot of things, and that you always carry with you the regret of the people that you've lied to, and people that you've hurt, and all the people that fell victim with the fraud, and you know, not being able to get closure from that too, because of, you know, privacy things, and like you carry that. But it's not all my blame to carry.” – Participant 3  “You can talk bad to yourself all you like but you're only hurting yourself. Don't talk to yourself like you’re a frigging loser, you should be dead, you should leave the kids alone, get out of their lives, all that negative stuff, no. So kindness to yourself, kindness to everybody else.” – Participant 13 |
| Rebuilding trust and reconnecting with others | “I want to be gamble free because I can prove to my sisters that I know there's more to – than gambling, and a holiday, or I want to give a good gift to my niece. I said to my sister I'll give her $500 when she has the baby. I've got it saved, she said oh it’s too much, I said don’t tell me what to do. Because they've been there for me. The two little grandkids, when they see me they're happy, I read them a book, give them a couple of dollars, and they’re happy to see me.” – Participant 1  “I got out of jail I was back to GA, and I’d mentor heaps of people from GA, I suppose counsel them is the word. I lived near a rooming house for prisoners that were just recently released, and I knew a couple of blokes up there and they used to send them down to me just to speak to them about reintegrating back into the world, and the dangers, stepping straight out of prison straight back into the environment they were in just before they went in, what can we do about that. That’s where strength and courage, responsibility and accountability comes into it.” – Participant 13  “When I was in deep addiction it was a nuisance, kids were a nuisance, I used to think like this. But now I have a connection with my - I have a great connection with my kids and a great connection with my grandkids. That is part and parcel of that honesty through love, kindness, to be proud of something else.” – Participant 13 |
| Reconnecting with hobbies and meaningful activities | “I went to the footy the other day. Me and my mate went to the Anzac match because I barrack for Richmond, so we went to that. That was a good night out, we didn’t get home until 11:30. That was real good, it helped me think, at the end of the day, more than gambling is enjoy sport or go out with family for meals or just go and see a movie.” – Participant 1  “Anyone that has got a gambling addiction or something, maybe they need to find something else in their life, whether that’s a dog, whether it’s a cat, whether it’s… or go to work for the Salvation Army three times a week. Find something good in your life. Don’t find something bad that’s going to cause you harm.” – Participant 13  “I have to do something that is going to make a difference. I have to do meaningful work or I’m not gonna work.” – Participant 10  “When you’re in the water with a mask on and the snorkel hanging out of your mouth. You can. You can drift in the water for hours, and not even realise how long you’ve been in there. I also, something I’d never done before I went down to the [location] to the back beaches. And there’s a few areas there where people dive off a cliff.” – Participant 4 |
| **Theme two:** **Navigating structural barriers and everyday triggers** | |
| Avoiding gambling environments | “So there were some side effects from giving up gambling. One of those was that driving home from work. Instead of taking me 15 minutes, it would take me an hour and 15 minutes because I was having to avoid any venues that I drove past. I couldn't physically walk past the venue if I went to the local main street. My legs would freeze. I'd start to shake. I think I was having a panic attack. So yeah, there was a physical reaction and they sort of lasted 6 to 12 months. And for probably 2 years I wasn't able to actually go into a venue I just didn't feel comfortable with that.” – Participant 4  “I don’t go to pubs because I can’t be in them, and it was hard because when we first got to the [venue] which is just down the road, the main bar wasn’t open, we had to sit in the pokies, and I’m like “Fuck”, you know, like I can do that, who knows how many times, I could do that 50 or 60 times but I’m telling you on the 61st time I’m busting, so I just can’t go to venues at all, you know?” – Participant 5  “Well, I've got a venue right on the corner here, and I haven't been in, not even for dinner. It's like having an alcoholic and putting a glass of alcohol in front of them. I don't want to put temptation in my way. Look. I might be strong enough, but I don't know, and I don't want to try it and I haven't had a need to. There's other restaurants. If I need to go to, and other food places, I don't need to go into a gambling venue to have dinner.” – Participant 6 |
| Experiences with gambling advertising as barriers to recovery | “I still get flooded every day you should see my spam folder, and I have unsubscribed to every single one I have. I've put in things to the ACCC, whatever it is and it doesn't make a God damn difference! They're all in on it. That's what's happening, because there's no other thing that explains it. But the persistence that would be persistence. What a waste of their time for me personally. Absolutely waste of the time.” – Participant 3  “Facebook has so many pop-ups that come up if you gamble on the horses or sport on TV, they're forever advertising all sorts of sports betting. At the actual games there's you know, those wraparound banners that they have around the ground that's always popping up. So yeah, it's really hard to avoid gambling. And I mean, when you have 100 and something gambling ads a day on free to air TV. You can't even turn on the TV cause they're all there. They're all on Facebook. They're all over the place, so it makes it really difficult.” – Participant 4  “I could imagine it could be quite a struggle for other people to see it advertised everywhere. Like those Sportsbet ads are crazy, the frequency, luring people in with humour. And I was into quite a few of those Sportsbet ads in the past and I understand their marketing appeal because people can sit there and identify with sport and have a chuckle, and humour is a brilliant way to hook people in subliminally I think. So I really feel for a lot of people out there who would be going through that.” – Participant 11 |
| Normalisation in everyday settings | “Sporting clubs need to take more responsibility in relation to the exposure for gambling opportunities. Now that also counts for those people who are trying to stop, people who are trying to stop. To walk into a club room and see that is an absolute trigger, so have some consideration. So get rid of all of that out of the club rooms. No gambling paraphernalia laying around, and understand that the gambling harm issue is huge, because a lot of people don't realise how huge it is, a lot of people still think it's a choice, they think it's greed and a choice. So [sporting] clubs must take more responsibility.” – Participant 13  “Even with playing footy and people would do footy multis, same game multis, so that's one. People would bring that up. Obviously, we play footy. We know the game so well. We think we do, obviously. So same game multis for footy, they would pop up. People doing quadies and things like that on a big day. So everyone's chipping in to do a quadie or a syndicate. So in that, there's obviously culture with that happening at sporting clubs and things like that. Another one, my football club that I play for our biggest sponsor is a sporting club, a sporting venue with pokies and a TAB. There's actually even some, here's another one that's actually baffling, I don’t know how I'd go with this, but there's actually footy clubs and netball clubs that have pokies as their social rooms. They've got pokies in their social rooms because it's a part of their club.” – Participant 12  “You can do it on the toilet. You can do it in church. You can do it at work. You can do it on auto spin. You know that you don't even need to be on the God damn computer or God damn phone. You could put it on auto spin and it'll spin it for you.” – Participant 3 |
| **Theme three:** **Living with grief, loss, and ongoing vulnerability in recovery** | |
| Recovery as a continuous battle | “I did have one lapse when my father in law passed away last year. So grief is a trigger. Obviously yeah, but it’s not, It's not in me anymore. I don't get urges to go. It was about 2 weeks and a week and a half I gambled did some pretty bad damage in that time, but my partner is loving and supportive, and kind and understanding, and I have all my supports.” – Participant 3  “I think I gambled for about 20 years. It was all – I can’t really pinpoint why; all I know is that it harmed, right. It was around the time that my little sister died that I felt very alone. And so I would find refuge in the Tabaret you know. Just to get away from all my problems I guess. And then when I was addicted well it was very hard to stop. I had a little bit of money, I blew all that. And then I just found it very difficult to survive and still am finding it difficult, even though a year I haven’t gambled.” – Participant 8  “I’d come quite a long way. But when I did move into the place, it was almost like I had a second kind of… Well, not almost. I did have a second kind of grieving about my mum. It was really, really bad, but I didn’t gamble then. I did drink, and then I sought help again for that.” – Participant 9  “Well there are [venues] within walking distance. I got so many, and I walk past them. When mum died. I was really sad, and every now and then I still get sad I think there was a couple of times where I had itchy fingers. Yeah, I just came home and kept myself busy.” – Participant 6 |
| Moments of change and new beginnings | “I attempted to take my life again. This time I was knocked unconscious, and I don't know how long I was unconscious for. But when I did regain consciousness, my family, my kids, they just rushed to my head, and they were just spinning around in my head, and I decided there and then that I really need to change something. I had to do something because I couldn't keep going the way I was.” – Participant 4  “Really dark places. Probably the best thing, my parents died, made me see the light, it’s cruel to say but see I don't want to waste the rest of my life in a TAB or a racetrack or at a pub, so it's now or never.” – Participant 1 |
